# Surgical indication for and desirable outcomes of conversion surgery in patients with initially unresectable pancreatic ductal adenocarcinoma

**DOI:** 10.1002/ags3.12295

**Published:** 2019-10-29

**Authors:** Sohei Satoi, Tomohisa Yamamoto, So Yamaki, Tatsuma Sakaguchi, Mitsugu Sekimoto

**Affiliations:** ^1^ Department of Surgery Kansai Medical University Hirakata‐City Japan

**Keywords:** conversion surgery, overall survival, resectability, surgical indication, unresectable pancreatic ductal adenocarcinoma

## Abstract

Aim of this review is to propose an acceptable surgical indication for conversion surgery in patients with initially unresectable (UR) pancreatic ductal adenocarcinoma (PDAC) by considering desirable outcomes, including resectability, overall survival (OS), and disease‐free survival (DFS). A comprehensive literature search of PubMed was conducted through July 15, 2019. Eligible studies were those reporting on patients with UR‐PDAC who underwent surgery. We excluded case reports with fewer than 10 patients, insufficient descriptions of survival data, and palliative surgery. When patients with UR‐PDAC with no progression after chemo(radiation) therapy were offered surgical exploration, resectability and median survival time (MST) of those who underwent conversion surgery ranged from 20% to 69% (median, 52%) and from 19.5 to 33 months (median, 21.9 months), respectively. When conversion surgery was carried out in patients with expected margin‐negative resection or with clinical response by Response Evaluation Criteria In Solid Tumors (RECIST), resectability and MST ranged from 18% to 27% (median, 20%) and from 21 to 35.3 months (median, 30 months), respectively. Among patients who underwent conversion surgery based on clinical response and decreased CA19‐9 level after multimodal treatment, resectability and MST ranged from 2% to 24% (median, 4.1%) and from 24.1 to 64 months (median, 36 months), respectively. Decreased CA19‐9 level was a predictor of resectability, OS and DFS by multivariate analysis. In conclusion, decision‐making for conversion surgery based on clinical response and decreased CA19‐9 level after multimodal treatment may be appropriate. With regard to desirable outcomes of OS and DFS, conversion surgery may provide improved survival for patients with initial UR‐PDAC.

## Graphical abstract

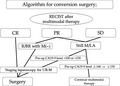

## 1 INTRODUCTION

Pancreatic ductal adenocarcinoma (PDAC) continues to have a dismal prognosis with a 5‐year survival rate of <5%, even in the modern era.^1^, ^2^ Most (70%–80%) patients with PDAC have unresectable (UR) disease, which is subclassified according to the status of distant metastasis—locally advanced disease (UR‐LA) and metastatic disease (UR‐M), such as distant organ metastasis and non‐regional lymph node metastasis. Recent implementation of new regimens, such as FOLFIRINOX^3^ and gemcitabine + nab‐paclitaxel,^4^ has provided better clinical response rates, ranging from 23% to 31.6%, and median survival time (MST), ranging from 8.5 to 12 months, even in patients with metastatic PDAC. Recently, conversion surgery, an additional surgery during multimodal therapy in patients with initial UR‐PDAC, has been introduced with the goal of prolonging short‐ and long‐term survival. Number of publications on conversion surgery has increased in recent years.^5^, ^6^, ^7^, ^8^, ^9^, ^10^, ^11^, ^12^, ^13^, ^14^, ^15^, ^16^, ^17^, ^18^, ^19^, ^20^, ^21^, ^22^, ^23^, ^24^, ^25^, ^26^, ^27^, ^28^, ^29^, ^30^ Several review articles^31^, ^32^, ^33^, ^34^, ^35^, ^36^ have reported high resectability rates, high margin‐negative resection rates, and high negative lymph node rates in patients who underwent conversion surgery with acceptable mortality and morbidity. MST in patients with initial UR‐PDAC who underwent conversion surgery was better than that of patients who did not undergo conversion surgery. However, most publications have described unclear surgical indications and varying rates of resectability, overall survival (OS), and disease‐free survival (DFS).^5^, ^6^, ^7^, ^8^, ^9^, ^10^, ^11^, ^12^, ^13^, ^14^, ^15^, ^16^, ^17^, ^18^, ^19^, ^20^, ^21^, ^22^, ^23^, ^24^, ^25^, ^26^, ^27^, ^28^, ^29^, ^30^ As evidence‐based guidelines for the management of UR‐PDAC are lacking, this review aims to propose an optimal surgical indication considering desirable outcomes of conversion surgery, with special consideration to resectability, OS, and early recurrence rate.

## 2 METHODS

### 2.1 Search strategy and data sources

Identification of eligible studies was carried out through a search of PubMed (MEDLINE) through 15 July 2019. The following search terms were used: “(unresectable pancreatic ductal adenocarcinoma OR unresectable pancreatic cancer) AND (pancreatectomy OR surgical resection)”. Finally, the reference lists of eligible studies were assessed manually to detect any potentially relevant articles (“snowball” procedure).

### 2.2 Inclusion and exclusion criteria

Eligible studies were those reporting on patients with histologically confirmed unresectable PDAC who underwent surgery after multimodal therapy, including chemotherapy/radiation therapy. Exclusion criteria were as follows: (i) irrelevant studies, (ii) editorials and letters to the editor, (iii) non‐English articles, (iv) case reports including fewer than 10 patients undergoing surgical resection, (v) insufficient description of survival data, and (vi) studies involving treatment mainly by ablative or non‐surgical technologies.

### 2.3 Data extraction and tabulation

Two authors (S.S. and T.Y.) conducted data extraction. Variables of interest included general study characteristics (eg, study period, study design, number of patients, resectability), regimens of multimodal therapy and percentages of patients who received them, surgical indication, OS and DFS, and predictive factors for surgical outcome. Data were tabulated when possible. Discordant judgment was resolved by discussion and consensus.

## 3 RESULTS

### 3.1 Article selection and study demographics

Following the initial algorithm and the successive steps of the selection process, including screening of the titles and abstracts, six review articles^31^, ^32^, ^33^, ^34^, ^35^, ^36^ and 26 original articles,^5^, ^6^, ^7^, ^8^, ^9^, ^10^, ^11^, ^12^, ^13^, ^14^, ^15^, ^16^, ^17^, ^18^, ^19^, ^20^, ^21^, ^22^, ^23^, ^24^, ^25^, ^26^, ^27^, ^28^, ^29^, ^30^ including two phase II studies and one prospective cohort study, were selected for this review. Finally, 26 articles reported surgical outcomes after conversion surgery (Table 1).^5^, ^6^, ^7^, ^8^, ^9^, ^10^, ^11^, ^12^, ^13^, ^14^, ^15^, ^16^, ^17^, ^18^, ^19^, ^20^, ^21^, ^22^, ^23^, ^24^, ^25^, ^26^, ^27^, ^28^, ^29^, ^30^ Seventeen articles showed a surgical indication for conversion surgery in patients with initial UR‐PDAC.^10^, ^11^, ^13^, ^15^, ^17^, ^19^, ^20^, ^21^, ^22^, ^23^, ^24^, ^25^, ^26^, ^27^, ^28^, ^29^, ^30^ Fifteen articles^7^, ^9^, ^10^, ^12^, ^13^, ^14^, ^16^, ^18^, ^19^, ^22^, ^23^, ^24^, ^25^, ^26^, ^30^ were abstracted for investigation of predictive factors for resectability, OS and DFS.

**Table 1:** Publications reporting clinical outcomes in patients with initially unresectable pancreatic ductal adenocarcinoma who underwent conversion surgery

First author	Year of publication	Study design	Study period	Category of UR, %	Regimen of chemotherapy	No. of patients	No. of resections	Resectability, %	MST (mo)
Sa Cunha^5^	2005	Retro	98‐03	UR‐LA	FP + RT	61	13	21	28
Bickenbach^6^	2012	MCC	00‐09	UR‐LA	GEM‐based	NA	36	NA	30
Sadot^7^	2015	Retro	10‐13	UR‐LA	FFX	101	31	31	NR
Marthey^8^	2015	Prosp	10‐12	UR‐LA	FFX	77	28	36	24.9
Bednar^9^	2017	Retro	10‐14	UR‐LA	FFX/ GnP 67/ others 34	92	19	20	32
Reni^10^	2017	Retro	02‐16	LA68/BR32	Gem‐based	223	61	27	30
Gemenetzis^11^	2019	Retro	13‐17	UR‐LA	FFX 50	415	84	20	35.3
Lee^12^	2018	Retro	12‐16	UR‐LA	FFX	64	15	23	>40, NR
Veldhuisen^13^	2018	Retro	13‐15	LA93/BR7	FFX 89	54	11	20.3	29
Yoo^14^	2019	Retro	05‐17	LA52/BR48	FFX 49	NA	135	NA	29.7
Murphy^15^	2019	Phase II	13‐18	UR‐LA	FFX + losartan + RT	49	34	69	33
Michelakos^16^	2019	Retro	11‐16	LA51/BR49	FFX	141 surgically explored	110	NE	37.7
Rangelova^17^	2019	Retro	10‐17	LA85/BR14	FFX 35	156	52	33	BR32, LA22
Satoi^18^	2013	Retro	01‐09	UR‐LA/M	Multi‐regimen	159^a^	58	NA	39.7
Hackert^19^	2016	Retro	01‐15	UR‐LA/M	FFX	125	76	61	21
					GEM + RT	322	150	47	21.5
					Others	128	66	52	19.5
Opendro^20^	2014	Retro	06‐13	UR‐LA/M	Multi‐regimen	130	13	10	36
Asano^21^	2018	Retro	07‐17	UR‐LA/M	Multi‐regimen	NA	34	NA	64
Byun^22^	2019	Retro	11‐17	BR20/ LA40/ M40	FFX	337	61	18	21
Heger^23^	2019	Retro	01‐17	LA73/M27	FFX 32	318	165	52	23
Natsume^24^	2019	Retro	12‐17	LA25/M75	GnP 29/ FFX 10	434	18	4.1	>36, NR
Klaiber^25^	2019	Retro	06‐17	LA73/M26	FFX 33	NA	280	NA	24.1
Crippa^26^	2016	Retro	03‐13	UR‐liver	Multi‐regimen	127	11	8.7	39
Wright^27^	2016	Retro	08‐13	UR‐M	FFX 61	1147	23	2	34.1
Satoi^28^	2017	Phase II	12‐15	UR‐PM	S‐1 + i.v./i.p. PTX	33	8	24	26
Frigerio^29^	2017	Retro	07‐15	UR‐M	FFX 67	535	24	4.5	56
Tanaka^30^	2019	Retro	11‐17	UR‐M	FFX	101 surgically explored	43	43	21.9

Abbreviations: BR, borderline resectable; FFX, FOLFIRINOX; FP, 5FU + CDDP; GEM, gemcitabine; GnP, GEM + nab‐paclitaxel; LA, locally advanced; M, metastasis; MCC, matched‐case control study; MST, median survival time; NA, not available; NE, not examined; NR, not reported; PM, peritoneal metastasis; prosp, prospective study; PTX, paclitaxel; Retro, retrospective study; RT, radiation therapy; UR, unresectable.

a Patients with clinical response for ≥6 mo after multimodal therapy.

### 3.2 Outcomes of conversion surgery

Several articles reported that conversion surgery could be carried out safely despite a high incidence of portal vein or arterial resection, ranging from 8% to 81%.^5^, ^6^, ^7^, ^8^, ^9^, ^10^, ^11^, ^12^, ^13^, ^14^, ^15^, ^16^, ^17^, ^18^, ^19^, ^20^, ^21^, ^22^, ^23^, ^24^, ^25^, ^26^, ^27^, ^28^, ^29^, ^30^ Mortality and morbidity ranged from 0% to 7% and from 14% to 89%, respectively.^5^, ^6^, ^7^, ^8^, ^9^, ^10^, ^11^, ^12^, ^13^, ^14^, ^15^, ^16^, ^17^, ^18^, ^19^, ^20^, ^21^, ^22^, ^23^, ^24^, ^25^, ^26^, ^27^, ^28^, ^29^, ^30^ Several studies showed high resectability rates (UR‐LA, 20% to 69%;^5^, ^6^, ^7^, ^8^, ^9^, ^10^, ^11^, ^12^, ^13^, ^14^, ^15^, ^16^, ^17^ UR‐LA/M, 4.1% to 61%;^18^, ^19^, ^20^, ^21^, ^22^, ^23^, ^24^, ^25^ UR‐M, 2% to 43%^26^, ^27^, ^28^, ^29^, ^30^), high margin‐negative resection rates (UR‐LA, 55% to 89%; UR‐LA/M, 27% to 89%; UR‐M, 51% to 91%), and high negative lymph node rates (UR‐LA, 38% to 83%; UR‐LA/M, 29% to 89%; UR‐M, 50% to 63%) in patients who underwent conversion surgery. MST in patients with initial UR‐LA,^5^, ^6^, ^7^, ^8^, ^9^, ^10^, ^11^, ^12^, ^13^, ^14^, ^15^, ^16^, ^17^ with LA/M,^18^, ^19^, ^20^, ^21^, ^22^, ^23^, ^24^, ^25^ and with M^26^, ^27^, ^28^, ^29^, ^30^ ranged from 24.9 to >40 months, 19.5‐64 months, and 21.9‐56 months, respectively. Although surgical indication and resectability varied, MST did not seem to vary according to resectability status.

### 3.3 Surgical indication for and resectability of conversion surgery

Seventeen articles reported a surgical indication for conversion surgery in patients with initial UR‐PDAC.^10^, ^11^, ^13^, ^15^, ^17^, ^19^, ^20^, ^21^, ^22^, ^23^, ^24^, ^25^, ^26^, ^27^, ^28^, ^29^, ^30^ From a review of the articles, surgical indication seemed to be classified into broad and strict criteria. Because of the lack of accuracy of current imaging modalities to predict the resectability of UR‐PDAC, some authors advocated that patients with UR‐PDAC with no progression after chemo(radiation) therapy should be offered surgical exploration in the absence of reliable predictors of resectability.^13^, ^15^, ^17^, ^19^, ^23^, ^30^ Resectability and MST of patients who underwent conversion surgery ranged from 20% to 69% (median, 52%) and from 19.5 to 33 months (median, 21.9 months), respectively.^13^, ^15^, ^17^, ^19^, ^23^, ^30^ Other authors recommended conversion surgery in patients who were expected to have margin‐negative resection or with clinical response by Response Evaluation Criteria In Solid Tumors (RECIST).^10^, ^11^, ^22^ Resectability and MST ranged from 18% to 27% (median, 20%) and from 21 to 35.3 months (median, 30 months), respectively.^10^, ^11^, ^22^ Moreover, the majority of institutions carried out conversion surgery based on clinical response defined by RECIST and decreased CA19‐9 level after multimodal therapy.^20^, ^21^, ^24^, ^25^, ^26^, ^27^, ^28^, ^29^ Resectability and MST ranged from 2% to 24% (median, 4.1%) and from 24.1 to 64 months (median, 36 months), respectively. 20, 21, 24, 25, 26, 27, 28, 29 In patients with metastatic PDAC, the surgical indication seemed to be stricter; major biochemical and radiological response (decreased tumor marker, tumor shrinkage of primary and metastatic site to single liver metastasis remaining or disappearance of peritoneal metastasis on staging laparoscopy).^27^, ^28^, ^29^ Resectability ranged from 2% to 24%, and MST ranged from 26 to 56 months. Very recently, Tanaka et al^30^ reported surgical outcomes under relatively broad surgical indications for metastatic PDAC, such as a maximum of six metastatic lesions, no tumor progression, and technically resectable disease. Resectability was 43%, and MST was 21.9 months.

### 3.4 Predictive factors for resectability and overall survival

#### 3.4.1 Resectability

Five articles identified prognostic factors for resectability in patients with initial UR‐PDAC.^7^, ^12^, ^13^, ^23^, ^30^ Four of them found that decreased CA19‐9 level was a predictor of resectability.^7^, ^13^, ^23^, ^30^ Sadot et al^7^ reported clinical outcomes (31% resectability) of 101 patients with stage III PDAC treated with FOLFIRINOX. This study showed that radiographic response and reduction in serum CA19‐9 level were associated with resectability by univariate analysis. van Veldhuisen et al^13^ reported that 11 of 54 patients with UR‐LA PDAC after chemotherapy (mostly FOLFIRINOX) were surgically resected (20.3%). A decrease in CA19‐9 level ≧30% was associated with improved survival (22.4 vs 12.7 months, *P* = .02) which was better than RECIST‐regression criteria. Lee et al^12^ also reported that 15 of 64 patients (23%) underwent surgical resection after initiation of FOLFIRINOX. A full dose of FOLFIRINOX was the only predictive factor for resectability. In the Heidelberg group, Heger et al^23^ reported that a CA19‐9 level <91.8 and a CA19‐9 ratio <0.407 were independent predictors of resectability in 318 patients with UR‐PDAC. Tanaka et al^30^ conducted a retrospective study in 101 metastatic patients, and 43 patients underwent pancreatectomy combined with metastasectomy. In this cohort, shrinkage of the primary tumor ≧0.5 and post‐chemotherapy CA19‐9 level <150 U/mL were independent prognostic factors for resectability.^30^

#### 3.4.2 Overall survival and disease‐free survival

Eleven articles reported prognostic factors for OS in patients with initial UR‐PDAC.^9^, ^10^, ^14^, ^16^, ^18^, ^19^, ^22^, ^24^, ^25^, ^26^, ^30^ Eight of them found that decreased CA19‐9 level was a prognostic factor for OS.^10^, ^16^, ^19^, ^22^, ^24^, ^25^, ^26^, ^30^ Three articles found that CA19‐9 response was a prognostic factor for PFS.^14^, ^16^, ^25^

Reni et al^10^ reported that 61 of 223 patients with UR‐LA/borderline resectable (BR) underwent surgical resection. Multivariate analysis showed that Karnofsky performance status, baseline T3/4, surgery, and CA19‐9 response were prognostic factors for OS. Michelakos et al^16^ reported that surgical resection was carried out in 110 patients of 141 patients who were surgically explored after FOLFIRINOX. Charlson Comorbidity Index of 0 or 1, CA19‐9 level ≦100 U/mL, tumor size ≦30 mm, and pathological tumor size ≦25 were prognostic factors for OS, and CA19‐9 level ≦100 and a time interval less than 8 months between initial treatment and surgical resection were prognostic factors for DFS. In a national audit conducted by the Japanese Society of Hepato‐Biliary‐Pancreatic Surgery, among 58 patients who underwent conversion surgery, it was found that a time interval of 8 months or longer between initial treatment and surgical resection was closely associated with improved survival.^18^ Hackert et al^19^ in the Heidelberg group compared OS in 575 patients with radiographically defined UR‐LA including occult distant organ metastasis according to type of chemotherapy such as FOLFIRINOX (n = 125), gemcitabine + radiation (n = 322), and other regimens (n = 128). The most effective treatment option was FOLFIRINOX, with a secondary resection rate of 61%. They also showed that FOLFIRINOX, surgical resection, CA19‐9 level <400, LA, and age younger than 70 years were prognostic factors for OS. Byun et al^22^ reported that 61 of 337 patients with initial UR‐PDAC underwent surgical resection after FOLFIRINOX induction. In this study, BR + LA versus metastasis, partial response + stable disease versus progressive disease, surgical resection and decreased CA19‐9 level were prognostic factors for OS. Natsume et al^24^ reviewed the clinical course of 434 patients with initial UR‐PDAC, and 18 patients (4.1%) underwent conversion surgery with a strict surgical indication. Proceeding to conversion surgery, albumin level, log_10_ (CA19‐9), log_10_ (tumor size), CA19‐9‐lowering rate, and tumor size‐lowering rate were predictive factors for OS. Klaiber et al^25^ in the Heidelberg group reported that preoperative CA 19‐9 levels ≧100 U/mL, lymph node involvement, M1 stage, and vascular infiltration were each independently associated with poor prognosis in 280 patients with initial UR‐PDAC who underwent surgical resection. Preoperative serum CA 19‐9 level was a prognostic factor for DFS. Bednar et al^9^ reported that radiation, ≧2 lines of chemotherapy, and surgery were significant independent prognostic factors for OS in 92 patients with initial UR‐LA (20% resectability). However, CA19‐9 level was not included in the multivariate analysis. In 135 resected patients, Yoo et al^14^ reported that prognostic factors for OS were age <65 years and partial response on RECIST criteria, and that prognostic factors for DFS were decreased CA19‐9 level and no vein resection.

Two of five articles^26^, ^27^, ^28^, ^29^, ^30^ dealing with UR‐M reported prognostic factors for OS and DFS. Crippa et al^26^ reported that chemotherapy with multiple agents, surgical resection, >5 liver metastases, and CA 19.9 reduction to <50% of the baseline value were prognostic factors for OS in 127 patients with liver metastasis (8.7% resectability). In the Heidelberg group, Tanaka et al^30^ reported that surgical resection was carried out in 43 of 101 patients who had a maximum of six metastatic lesions and no tumor progression after FOLFIRINOX induction from 2011 to 2017. Post‐chemotherapy CA19‐9 level <150 U/mL and lymph node ratio <0.1 were prognostic factors for OS.

## 4 DISCUSSION

On imaging studies, the majority of PDAC is classified as UR disease. The 5‐year OS rate remains less than 10% as a result of the high proportion of UR‐PDAC. In past decades, there have been several developments in the treatment of UR‐PDAC. Implementation of modern chemotherapy regimens, such as FOLFIRINOX and gemcitabine + nab‐paclitaxel, has provided better MST of 8‐12 months, even in metastatic PDAC,^3^, ^4^ and has led to the possibility of converting UR disease to resectable disease in patients with favourable response during multimodal therapy. Generally, an upfront surgical approach has not been justified in patients with UR‐PDAC due to the high frequency of mortality and morbidity and poor prognosis. Conversion surgery provided favorable outcomes of a high proportion of margin‐negative resection and negative lymph node metastasis, resulting in improved MST ranging from 19.5 to 64 months in patients with initial UR‐PDAC.^7^, ^9^, ^10^, ^12^, ^13^, ^14^, ^16^, ^18^, ^19^, ^22^, ^23^, ^24^, ^25^, ^26^, ^30^ When patients were selected after a favorable response to anticancer treatment followed by conversion surgery, MST did not seem to differ according to resectability status. Even in patients with M‐PDAC, conversion surgery should be considered if they fit the surgical indication. Articles from Japan evaluated clinical outcome of conversion surgery in patients with initial UR‐LA and UR‐M PDAC, but some articles from other countries evaluated clinical outcome of conversion surgery in patients with initial UR‐LA and UR‐M as well as BR‐PDAC, because OS in patients with BR‐PDAC who underwent surgical resection following multimodal treatments has been reported to be similar to that in patients with UR‐LA PDAC.^17^

Actual resectability of conversion surgery is difficult to quantify due to varying populations in each institution, because candidates for conversion surgery are generally centralized in high‐volume centers. There is a possibility of publication bias or patients’ selection bias in the selected articles. In a review article, Suker et al^37^ reported a resectability rate of 28% after FOLFIRINOX ± radiation therapy in patients with UR‐LA. Resectability after FOLFIRINOX ranged from 20% to 69% in patients with UR‐LA in 10^7^, ^8^, ^9^, ^17^ of 13 articles,^5^, ^6^, ^7^, ^8^, ^9^, ^10^, ^11^, ^12^, ^13^, ^14^, ^15^, ^16^, ^17^ 4.1% to 61% in UR‐LA/M in two^19^, ^22^ of eight articles,^18^, ^19^, ^20^, ^21^, ^22^, ^23^, ^24^, ^25^ and 2%‐43% in UR‐M in three^27^, ^29^, ^30^ of five articles.^26^, ^27^, ^28^, ^29^, ^30^ Rates of resectability after FOLFIRINOX seemed to be higher relative to other regimens.

In the present review, the surgical indication for conversion surgery remains unclear, and it differed in each institution. One institution had strict criteria, as follows: tumor shrinkage to R/BR status, decreased levels of tumor marker, maintenance of performance status, and a long interval between initial treatment and surgical resection.^11^, ^20^, ^21^, ^24^, ^28^ Strict criteria may lead to lower resectability but longer OS as a result of patient selection. Another institution had a relatively broad surgical indication of no tumor progression for UR‐LA, or metastasectomy of a maximum of six metastatic lesions for UR‐M.^13^, ^15^, ^17^, ^19^, ^23^, ^30^ Broad criteria may be associated with higher resectability but shorter OS due to the risk of early recurrence after conversion surgery.

Optimal selection criteria for surgical exploration or resection remains controversial in patients with initial UR‐PDAC. Four of five articles that identified predictors of resectability showed that decreased CA19‐9 level was a predictor.^7^, ^13^, ^23^, ^30^ Eight of 11 articles identifying predictors of OS also showed decreased CA19‐9 level as a prognostic factor for OS.^10^, ^16^, ^19^, ^22^, ^24^, ^25^, ^26^, ^30^ All three articles reporting prognostic factors for DFS showed that CA19‐9 response was a prognostic factor.^14^, ^16^, ^25^ Among them, the Heidelberg group clearly showed that a post‐chemotherapy CA19‐9 level <100 U/mL was a favorable prognostic factor for OS and a post‐chemotherapy CA19‐9 level ≧100 U/mL was a predictor of poor DFS in 280 patients with initial UR‐PDAC, including BR in 6%, UR‐LA in 68%, and UR‐M (oligometastasis) in 26%.^25^ They also showed that a post‐chemotherapy CA19‐9 level <150 U/mL was a favorable prognostic factor for OS as well as DFS in 101 patients with UR‐M undergoing exploratory surgery (43 patients resected).^30^ In contrast, Rangelova et al^17^ reported that for all preoperative values of CA19‐9, surgical resection had a positive impact on survival. They concluded that all patients with BR/LA‐PDAC who did not progress during multimodal therapy should be considered for surgical resection, irrespective of the type or dose of regimen given. Higher levels of CA19‐9 should not be considered an absolute contraindication for resection.^17^ Although it is still controversial, several articles reported that a decreased CA19‐9 level after multimodal therapy was a reliable predictive factor for resectability, OS, and DFS.^7^, ^10^, ^13^, ^14^, ^16^, ^19^, ^22^, ^23^, ^24^, ^25^, ^26^, ^30^ Tsai et al also suggested that a decrease in CA19‐9 level following systemic therapy was a useful marker for treatment success, even in patients with localized PDAC.^38^ Thus, decision‐making for conversion surgery based on clinical response defined by RECIST and decreased CA19‐9 level after multimodal therapy may be appropriate.

**Figure 1:**
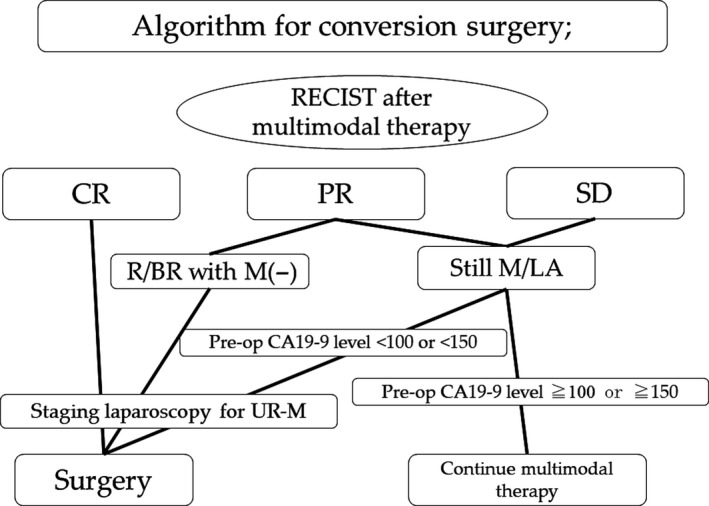
Algorithm for patients with unresectable pancreatic ductal adenocarcinoma who are re‐evaluated during multimodal therapy. BR, borderline resectable; CR, complete response; PR, partial response; R, resectable; RECIST, Response Evaluation Criteria In Solid Tumors; SD, stable disease; UR‐LA, unresectable locally advanced pancreatic ductal adenocarcinoma; UR‐M, unresectable metastatic pancreatic ductal adenocarcinoma

When patients with initial UR‐PDAC experience significant tumor shrinkage (complete or partial response) to R/BR status, decision‐making for conversion surgery is easy. However, decisions are still controversial in patients showing stable disease after multimodal therapy, because it is difficult to differentiate whether viable tumor tissue is present on contrast‐enhanced CT imaging.^16^, ^19^ In these situations, CA19‐9 level <100 U/mL or 150 U/mL in UR‐PDAC can be a reliable marker for conversion surgery (Figure 1). Moreover, use of diffusion‐weighted magnetic resonance imaging (MRI)^39^ or positron emission tomography (PET)‐CT scans^40^ may aid in selecting patients for conversion surgery. As occult liver or peritoneal metastasis is not sometimes accurately detected on CT imaging, staging laparoscopy should be done for evaluating the presence of occult distant metastasis before curative surgery, especially in patients with UR‐M (Figure 1).

### 4.1 Desirable outcomes for conversion surgery and future perspectives

Recent chemotherapy regimens, such as FOLFIRINOX and gem + nab‐PTX, provide better MST of 24.2 months in UR‐LA^37^ and 8.5‐12 months in UR‐M.^3^, ^4^ Although conversion surgery is expected to prolong survival, we should definitely recognize that the early recurrence rate (within 6 months) after conversion surgery is approximately 30%.^14^, ^16^, ^25^ In this situation, patients cannot expect a longer survival relative to non‐surgical patients, and conversion surgery may simply be a surgical injury for patients, because extensive pancreatectomy has a high risk of mortality and morbidity.^36^ The early recurrence rate should be decreased as much as possible in patients who undergo conversion surgery. From the prognostic point of view, desirable outcomes of an MST of 36 months in patients with UR‐LA and 24 months in patients with UR‐M and less than a 20% incidence of early recurrence after conversion surgery, but not high resectability, may be required for obtaining a survival benefit in the modern era. Therefore, the surgical indication for conversion surgery should be carefully decided in a multidisciplinary meeting and should be relatively limited according to radiological findings as well as the CA19‐9 level. van Veldhuisen et al^31^ have suggested that in addition to CA19‐9, other promising biomarkers, such as micro‐RNAs and circulating tumor DNA, may more accurately predict treatment response in UR‐PDAC.^41^, ^42^, ^43^ In the near future, reliable surrogate markers for predicting resectability, early recurrence, and favorable prognosis should be explored.

Moreover, the optimal timing between initial treatment and surgical resection, an accurate method to evaluate tumor remission, and the type/duration of multimodal therapy are still under investigation. Several prospective studies are now in progress.^35^, ^36^ Sustainable efforts will be required to prolong survival in patients with UR‐PDAC.

## 5 CONCLUSION

Number of candidates for conversion surgery is now increasing with the introduction of modern chemotherapy regimens; however, the actual clinical benefits of resection have not yet been fully investigated. Although conversion surgery can improve long‐term survival in patients with UR‐PDAC, the early recurrence rate should be recognized. There are still several problems to be resolved in this area, and prospective studies will be needed to explore the clinical benefit of conversion surgery. An appropriate surgical indication for achieving desirable outcomes can definitely provide further improved survival and early recurrence rates. Therefore, novel biomarkers predicting resectability, OS and DFS should be investigated in the near future.

## DISCLOSURE

Conflicts of Interest: Authors declare no conflicts of interest for this article.
